# Unveiling the occurrence of COVID-19 in a diverse Bangladeshi population during the pandemic

**DOI:** 10.3389/fpubh.2024.1363971

**Published:** 2024-05-31

**Authors:** Mahmud Hossain, Rezowana Mannan, Sohidul Islam, Laila A. Banu, Ahsan Rahman Jamee, Zahid Hassan, Sabrina Moriom Elias, Sanjan K. Das, A. K. Azad Khan

**Affiliations:** ^1^Department of Biochemistry and Molecular Biology, Laboratory of Neuroscience and Neurogenetics, University of Dhaka, Dhaka, Bangladesh; ^2^Dr. Farida Haq Memorial Ibrahim General Hospital, COVID-19 Diagnostic Laboratory, Gazipur, Bangladesh; ^3^Department of Virology, Institute of Epidemiology, Disease Control and Research (IEDCR), Dhaka, Bangladesh; ^4^Department of Biochemistry and Microbiology, School of Health and Life Sciences, North South University, Dhaka, Bangladesh; ^5^Department of Anatomy, Bangabandhu Sheikh Mujib Medical University, Dhaka, Bangladesh; ^6^Department of Statistics, University of Dhaka, Dhaka, Bangladesh; ^7^Department of Physiology and Molecular Biology, Bangladesh University of Health Sciences, Dhaka, Bangladesh; ^8^Department of Life Sciences, School of Environment and Life Sciences, Independent University, Dhaka, Bangladesh; ^9^Diabetic Association of Bangladesh, Dhaka, Bangladesh; ^10^National Professor, Bangladesh

**Keywords:** SARS-CoV-2, COVID-19, Public Health, RMG, Bangladesh

## Abstract

**Introduction:**

COVID-19 pandemic hit Bangladesh with relatively low intensity, unlike its neighbors India and European countries and USA.

**Methods:**

The present report included data of 8,480 individuals tested for COVID-19 RT-PCR of the workers and officials from readymade garments (RMG) industry in Chandra area in Gazipur. The present data looked into the clinic-demographic factors associated with the susceptibility of the condition.

**Result:**

The data elucidated the susceptibility of the individuals to SARS-CoV-2 based on age, gender, pre-existing health conditions, and the presence of symptoms. It was observed that individuals aged over 60 had the highest rate of COVID-19 positivity, and men exhibited a higher infection rate compared to women. Regardless of age, fever and cough were the most frequently reported symptoms. Two-thirds of the individuals included in this report appeared to be asymptomatic carriers. The prevalence of comorbidities among individuals who tested positive for COVID-19 was notably higher, and this exhibited a gender-specific pattern.

**Discussion:**

Although our study provides important epidemiological insights into the initial year of the pandemic among Bangladeshi populations, it can also add value for future drug and vaccine development. However, it is essential to acknowledge the limitations like - restriction of public movement, unavailability of vehicle yielding a selection bias, due to the lockdown conditions imposed owing to the pandemic and the diverse characteristics of the participants. The report emphasizes the significance of figuring out how age, gender, and underlying health conditions impact susceptibility to and transmission of COVID-19, thereby providing valuable insights for public health strategies and future research initiatives.

## 1 Introduction

The 21st century was introduced with the two most contagious global epidemics caused by the corona virus family, Severe Acute Respiratory Syndrome (SARS) in 2001–2003 and Middle East Respiratory Syndrome (MERS) in 2012–2015. The SARS was reported first in November 2002 in Foshan, Guangdong, China, and spread to 29 countries worldwide, leaving 774 death cases (1, 2). Since the MERS was first isolated in 2012, 27 countries reported cases till 2019 with 858 deaths (3, 4). Afterward, several different species of corona virus have been sequenced and categorized into four major genera known as *Alphacoronavirus, Betacoronavirus, Gammacoronavirus and Deltacoronavirus* (5).

The corona virus genome is known as the largest (comprised of ~32 kbp) among the plus-strand (except retrovirus) RNA viruses (HCov-229E: 27,317 nt to MHV-A59: 31,357 nt). Their unique replication strategy is usually associated with various diseases in versatile range of hosts (6). In December 2019, in Wuhan city, China, the most pathogenic and transmissible, a novel member, SARS-CoV-2 (*Betacoronavirus*), caused an outbreak termed COVID-19 (Corona Virus Disease 2019) that turned into a global pandemic by 2020 (7). The SARS-CoV-2 is the seventh member among the corona virus family infecting humans (8) that is confirmed to be 96% identical to a bat corona virus (nonpathogenic to humans) by Whole Genome Sequencing (9, 10). The SARS-CoV-2 uses the human Angiotensin Converting Enzyme-2 (ACE II) as a cellular entry receptor, thus being infectious to the human (11, 12). This novel virus has caused the unprecedented outbreak of COVID-19 that infected cumulatively about 216 million people and nearly 4.5 million death cases by August 2021 worldwide (13). Several COVID-19 diagnostic procedures have been introduced targeting either ORF (Open Reading Frame) encoding 27 different non-structural proteins or other conserved sequences of major structural proteins such as the nucleocapsid protein (N), spike surface glycoprotein (S), membrane protein (M) and small envelope protein (E) (14, 15).

The principal mode of transmission of corona virus is respiratory droplets (coughing or sneezing) with close contact an infected person and possibly a long time exposure in a closed environment by aerosol transmission (16–18). A wide spectrum of clinical complications were identified in COVID-19 patients, and few of them could not have recognized any symptoms (asymptomatic) (10). In mild to moderate cases, the most observed symptoms were fever, cough, rhinorrhea (runny nose), pharyngalgia (sore throat), myalgia (muscle pain), headache, fatigue, dyspnea (shortness of breathing), ageusia (loss of taste), anosmia (loss of smell) and diarrhea. In particular, severe cases of COVID-19 were identified as critical conditions with the Acute Respiratory Distress Syndrome (ARDS) that require extended care in an Intensive Care Unit (ICU) with ventilation support, septic shock, coagulopathy, arrhythmias, thrombocytopenia, and multiple organ dysfunction syndrome (19, 20).

Chronological age is one of the most important predictors of COVID-19 disease severity, especially a reliable biomarker for vaccine design with the highest patient satisfaction (21). However, comorbidities, for instance, diabetes mellitus, hypertension, asthma, Cardio Vascular Disease (CVD), or renal diseases, are observed to be associated with higher mortality rates, indicating that the biological age is a more pertinent risk factor for disease severity than the chronological age (22, 23). Most of the studies have reported male-biased COVID-19 cases, in addition to hospitalization, ventilation support, and fatality rate measurement of critical conditions (24, 25).

In early March of 2020, the Institute of Epidemiology, Disease Control and Research (IEDCR) first identified three cases of SARS-CoV-2 in Bangladesh. The Government imposed a nationwide lockdown in late March of 2020 (26, 27). To keep the GDP (Gross Domestic Product) trending, private sectors, especially readymade garments industries (RMG), play a key role in earning foreign currency. In the lockdown situation, running the garment factories and maintaining the health and safety of the workers was a great challenge. Hence, following the WHO guidelines, a fully dedicated COVID-19 RT-PCR laboratory was established in May 2020, closest to the EPZ (Export Processing Zone) near the capital Dhaka in Bangladesh. A several contagious variants of SARS-CoV-2 evolved globally over the time, and classified by Whole Genome Sequencing process (28). Even after mass vaccination program, a swift surge of the delta variant, later replaced by the omicron variant of SARS-CoV-2 caused a fatal increase in hospitalization (29). Furthermore, different types sub-variant of the omicron (Eris, Pirola) are identified in symptomatic subjects (30, 31). Hence, it is obvious that the COVID-19 has not been fully eradicated.

There is a high chance to get the positive result even in randomly selected asymptomatic subject due to the frequent mutation(s) of the virus. And, these mutations can be compared with the demographic and clinical data of the COVID-19 for further effective vaccine or drug developments. Recently, it has been reported using the data of 2020–2021 that even after the treatment with COVID-19 convalescent plasma, the post covid complications has been reduced compared with the demographic and clinical characteristics but could not be absolutely eradicated (32). Here, we are presenting the random population-based screening data of COVID-19 from June 2020 to August 2021 which can be used as a baseline data to understand the characteristics and transmission of the disease in Bangladesh.

## 2 Materials and methods

### 2.1 Study design

With the approval of the ethical review committee of the Department of Biochemistry and Molecular Biology, University of Dhaka, we performed a cross-sectional and descriptive population-based study to understand the baseline characteristics of COVID-19 and its epidemiology among the population predominantly from Bangladeshi garments and industrial sectors. This study evaluates a well-designed comparison between the prevalence rate and the COVID-19-associated symptoms and comorbidities according to the gender of various age groups.

### 2.2 Sample and data collection

**Figure 1:**
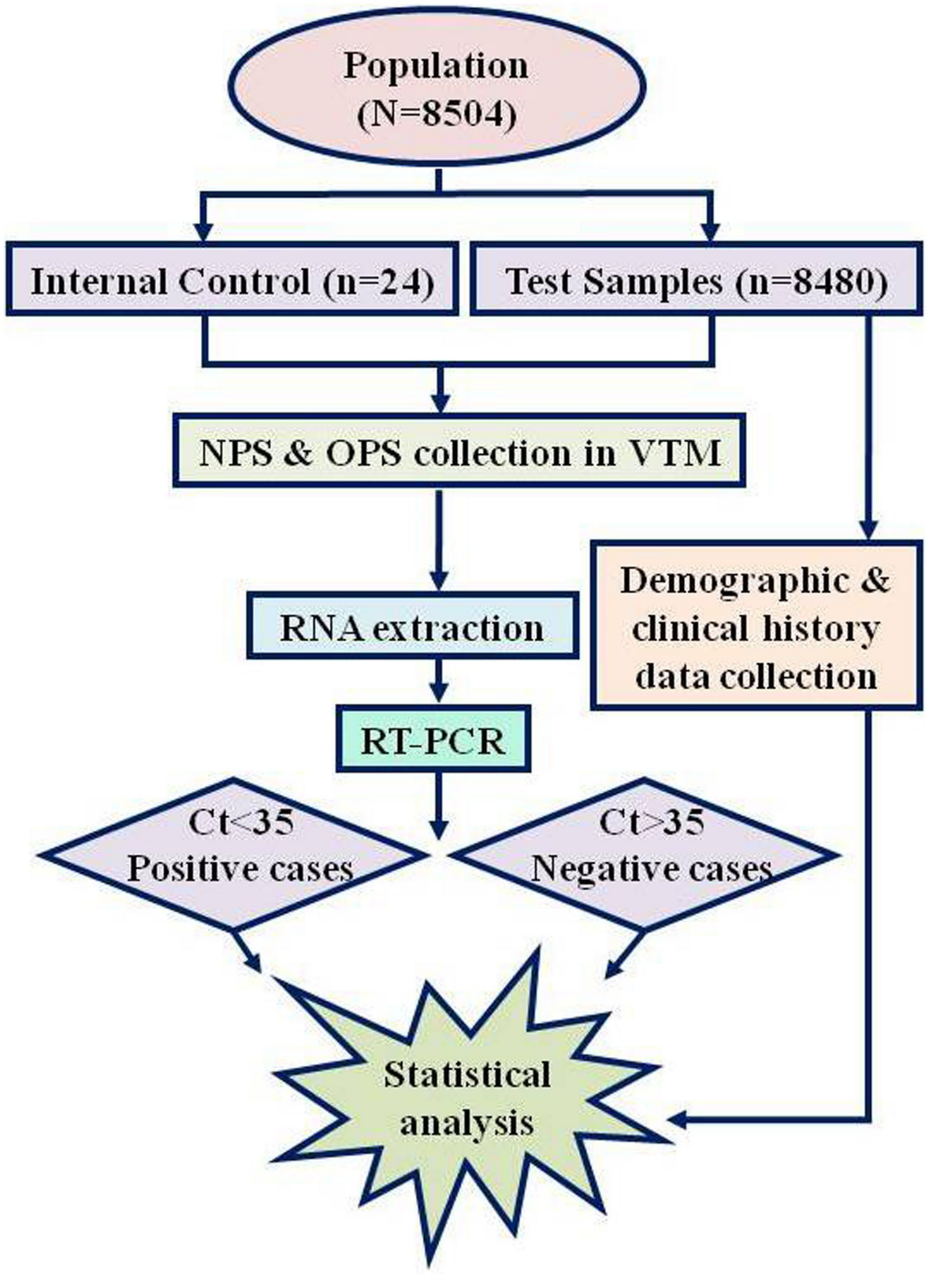
Flow diagram of COVID-19 study.

From June 2020 to August 2021 (15 months), 8504 samples were diagnosed at our laboratory (Dr. Farida Huq Memorial Ibrahim General Hospital COVID-19 Diagnostic Laboratory, Chandra, Gazipur). Of these 8,504 samples, 24 were run as the Internal Laboratory Quality Control Assay. Hence, data from 8,480 (eight thousand four hundred and eighty) samples were compiled for this study (Figure 1). The clinical information was uniformly recorded before the sample collection along with written consent of every individual for future research purposes. Accumulated information was scrutinized under several variables: demographic data (age, gender, and residence area), and clinical data (exposure history, symptoms, comorbidities, or any undergoing medication, including the COVID-19 vaccine). The whole population is divided into four age groups (Table 1) with additional subdivision by gender as male and female.

**Table 1:** Demographic characteristics of study participants with the prevalence of COVID-19 cases.

Characteristics	Study participants (*N* = 8,480)	COVID-19 Positive cases
	*N* (%)	(%)
Age (years)		
Below 20 years	710 (8.37)	19.30 (137/710)
21–40 Years	5,762 (67.95)	19.77 (1,139/5762)
41–60 Years	1789 (21.10)	24.09 (431/1789)
Above 61 Years	219 (2.58)	36.53 (80/219)
Sex		
Male	5,669 (66.85)	23.09 (1,309/5669)
Female	2,811 (33.15)	17 (478/2811)
Residence		
Gazipur	3,589 (42.32)	25.94 (931/3589)
Dhaka	4,334 (51.11)	16.5 (715/4334)
Tangail	274 (3.23)	40.51 (111/274)
Others	283 (3.34)	10.6 (30/283)
Symptoms		
Asymptomatic	5,738 (67.67)	13.96 (801/5738)
Symptomatic	2,742 (32.33)	35.96 (986/2742)
Comorbidity		
No	7,838 (92.43)	20.29 (1,590/7838)
Yes	642 (7.57)	30.69 (197/642)
COVID-19 vaccination		
Non-vaccinated	8,308 (97.97)	21.07 (1751/8308)
Vaccinated	172 (2.03)	20.93 (36/172)

Methods: Pearson chi-squared test was performed to test the measures of association between outcome and explanatory variables (*p* < 0.001).

### 2.3 Specimen collection

Following the instruction of the CDC (Centers for Disease Control and Prevention), both the Oropharyngeal Swab (OPS) and Nasopharyngeal Swab (NPS) samples were collected in the sterile VTM (Viral Transport Medium) and transported to the molecular laboratory maintaining the cold chain (33). Each sample tube was labeled with the unique Laboratory ID and sanitized before being delivered inside the double-door protected BSL-II lab via the dynamic pass-box.

### 2.4 Laboratory investigation

The Reverse Transcriptase Polymerase Chain Reaction (RT-PCR) is a gold standard molecular technique for identifying the SARS-CoV-2 from OPS and NPS samples. The test kit used was from Primer Design Ltd., UK. The RNA was extracted from the cell-free fluid samples either manually or automatically. The amplification kit was designed based on the TaqMan RT-PCR principle targeting two genes-Nucleocapsid (N) and ORF1ab regions, labeled probes with FAM (Fluorescein amidites, 465-510 nm) and ROX (6-carboxyl-X-Rhodamine, 576–601 nm) fluorescent dyes for the two amplicons. Following the literature on the detection kit, the amplification was performed using Bio-Rad CFX (USA).

For the validation of the RT-PCR, Negative Extraction Control (NEC) is required to produce cycle threshold, Ct < 30 in the VIC (2′-chloro-7′-phenyl-1, 4-dichloro-6-carboxyfluorescein) or HEX (Hexachloro fluorescein, 533–580 nm) channel, whereas Positive Control Template (PCT) dilution at 1.7 copies/μl produces Ct of 14–22 in the FAM channel. With this comparison, the Ct value of Internal Extraction Control (IEC) produced by the individual’s sample should be within the range of the Ct value peaked by the NEC (±6). The Ct < 35 was considered positive for SARS-CoV-2 (Figure 1). Internal Laboratory Quality Control Assay was regularly conducted and compared with another COVID-19-dedicated laboratory. Furthermore, for RT-PCR test result analysis validation, representative samples were delivered to the Institute of Epidemiology, Disease Control & Research (IEDCR), Mohakhali, Dhaka, every month who runs the national COVID-19 validation and optimization surveillance under the Ministry of Health and Family Welfare of Bangladesh. A concordance of ≥95% was obtained while samples were tested in a reference laboratory to ensure the quality of the test and result analysis parameters.

### 2.5 Data analysis

Pearson chi-squared test was performed to test the measures of association between outcome and explanatory variables, and a binary logistic regression model was performed to examine the unadjusted and adjusted effects of covariates on COVID-19 (34). All statistical analyses were performed using STATA 14.

## 3 Results

### 3.1 Demographic analysis

In the present study, both symptomatic and asymptomatic people randomly visited the laboratory requesting for COVID-19 test from different zones, mainly from Gazipur (42.32%), Dhaka (51.11%), Tangail (3.23%), and other areas (3.34%) of Bangladesh. Despite the majority of samples being gathered from the Dhaka region, the Gazipur area exhibited a higher COVID-19 prevalence, with Tangail recording the highest prevalence at 16.50, 25.94, and 40.51%, respectively (*p* < 0.001) (Table 1). Out of the samples received, approximately two-thirds were male (66.85%), and they exhibited a COVID-19 positivity rate of 23.09% (*p* < 0.001). In contrast, female participants, accounting for 33.15% of the samples, displayed a COVID-19 positivity rate of 17.00% (*p* < 0.001) (Table 1).

**Figure 2:**
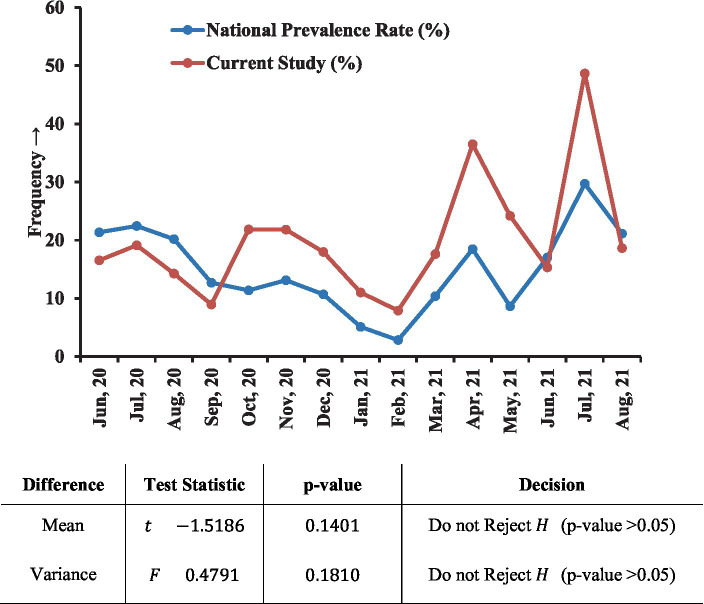
Prevalence of COVID-19 in the laboratory compared with the national scenario. Within the 15 months (June 2020–August 2021) study period, this graph showed a similar month-wise pattern to the national frequency. T-test and variance (F) tests were performed to check whether any difference exists between our laboratory data and the national case frequency rate where no significant difference was found.

The test-participants’ age spanned from 1.5 months to 95 years, with a mean age of 33.9 ± 11.8 years. They were categorized into four groups based on age, below 20 years (8.37%), 20–40 years (67.95%), 40–60 years (21.10%), and above 60 years (2.58%). The higher prevalence rate of COVID-19 was observed in individuals belong to higher age group; 19.30, 19.77, 24.09, 36.53%, respectively (*p* < 0.001) (Table 1). Nevertheless, the subjects randomly enrolled in our study within 15 months, showed similar prevalence patterns (month-wise) with the national prevalence rate (Figure 2).

After the initiation of mass COVID-19 vaccination on early February 2021 in Bangladesh, we recorded only 2.03% vaccinated cases, and the positivity rate was almost similar for both vaccinated (20.93%) and non-vaccinated (21.08%) subjects (Table 1).

### 3.2 Clinical characteristics of SARS-CoV-2 positive subjects

**Figure 3:**
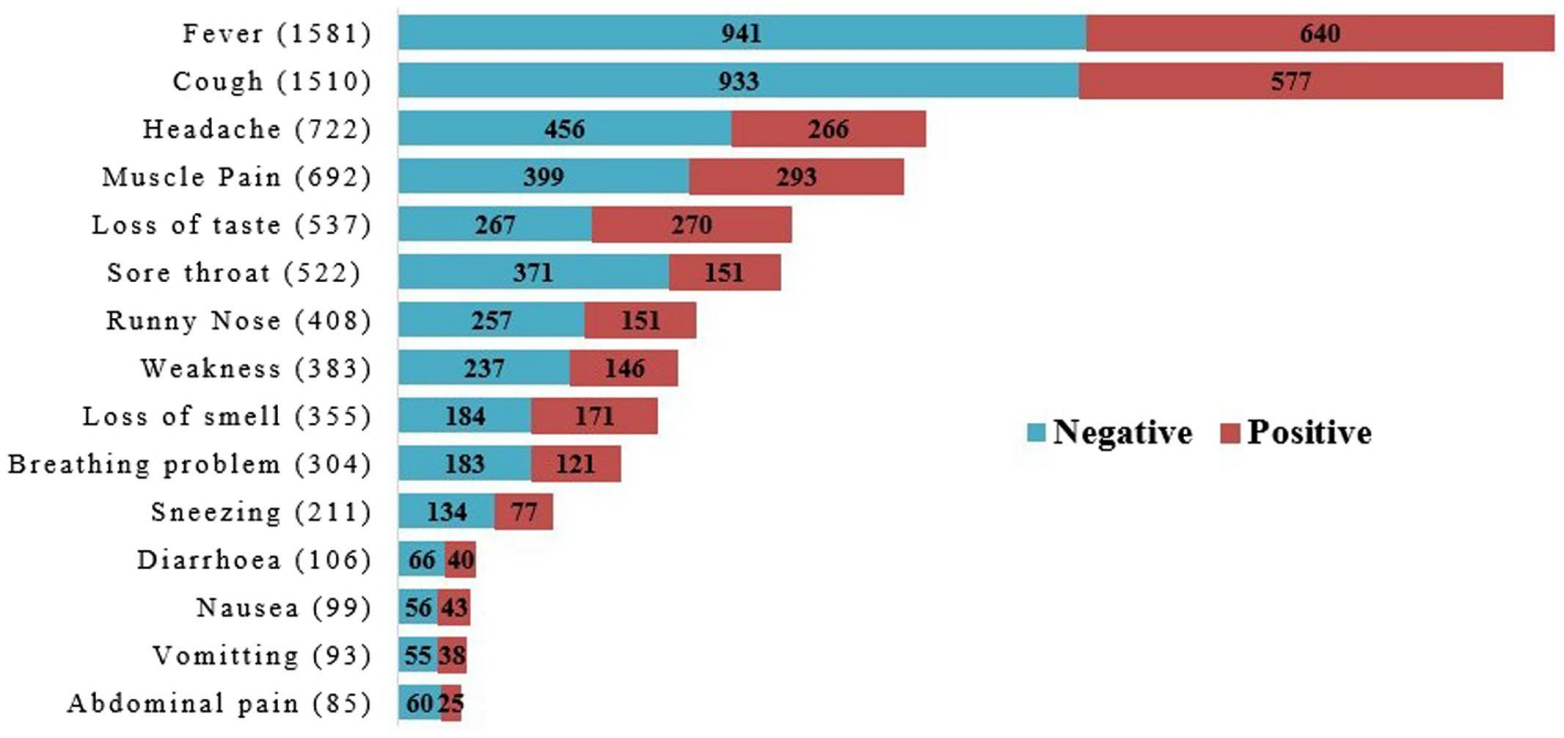
Symptoms associated with COVID-19 (both positive and negative cases) in study population.

Almost one-third of the total population (32.33%) showed mild to severe COVID-19-associated symptoms such as fever, cough, runny nose, headache, muscle pain, breathing problems, nausea, vomiting, abdominal pain, sore throat, diarrhea, loss of taste, loss of smell, and weakness (Table 1; Figure 3). Among these symptomatic population 35.96% were diagnosed as COVID-19 positive whereas 13.96% showed in asymptomatic subjects (*p* < 0.001) (Table 1).

Analyzing the symptoms associated with COVID-19, fever (57.66%) and cough (55.07%) were counted as the highest along with headache (26.33%) and muscle pain (25.23%) (Figure 3). Relatively lower number of subjects noticed two of the most relevant COVID-19 symptoms-loss of taste (19.58%), and loss of smell (12.95%) despite, around 50% cases were found positive for both symptoms (Figure 3). Rest of the nine symptoms occurred in about 30–40% positive cases, gradually as nausea (43.43%), vomiting (40.86%), weakness (38.12%), diarrhea (37.74%), runny nose (37%), sneezing (36.49%), breathing problem (39.80%), abdominal pain (29.41%) and sore throat (28.93%) (Figure 3).

### 3.3 Gender and comorbidities relating to COVID-19 cases

Gender variation showed a noticeable difference in RT-PCR results, presence of symptoms, and pre-existing medical history (Tables 1, 2). With the observation of the RT-PCR results of the whole population (N=8480), two-thirds of positive samples were from male individuals (n=1309, 15.44%) compared with the females (n=478, 5.64%) (Table 1). Comorbidities were reported in 7.57% of the total population, wherein 30.69% were tested as COVID-19 positive (Table 1). The reported comorbidities were asthma, diabetes, hypertension, cardiovascular diseases, chronic renal diseases, immunocompromised, neural, and other diseases (constipation, typhoid, spinal pain, jaundice, sinusitis, major surgery, etc.).

**Table 2:** Different comorbidities in gender variation.

Comorbidities (*n* = 642, 7.57%)**		Male (*n* = 483, 75.23%)	Female (*n* = 159, 24.76%)
Diabetes mellitus	(*n* = 303, 47.20%)	237 (78.21%)	66 (21.78%)
Hypertension	(*n* = 299, 46.57%)	237 (79.26%)	62 (20.74%)
Asthma	(*n* = 117, 18.22%)	79 (67.52%)	38 (32.48%)
CVD	(*n* = 53, 8.26%)	46 (86.79%)	7 (13.21%)
Renal disease	(*n* = 21, 3.27%)	16 (76.19%)	5 (23.81%)
Cancer	(*n* = 13, 2.02%)	6 (46.15%)	7 (53.85%)
Immunocompromised	(*n* = 7, 1.09%)	2 (28.57%)	5 (71.43%)
Ulcer	(*n* = 4, 0.62%)	3 (75%)	1 (25%)
Tonsillitis	(*n* = 3, 0.47%)	0	3 (100%)
Neural disease	(*n* = 3, 0.47%)	2 (66.67%)	1 (33.33%)
Hypothyroidism	(*n* = 2, 0.31%)	1 (50%)	1 (50%)
Others	(*n* = 27, 4.21%)	13 (48.15%)	14 (51.85%)

^**^Percentages and totals are based on respondents.

(Comorbidities are dichotomy groups tabulated at value 1).

Among the comorbidities individuals, diabetes mellitus (47.19%) and hypertension (46.57%) upheld the same highest frequency, followed by asthma (18.22%). Male biased cross tabulation was analyzed in each of the parameters: 78.21% diabetes, 79.26% hypertension, 67.52% asthma, 86.79% cardiac diseases, and 66.67% neural diseases showed being doubled than the female respondent. Immunocompromised patients (1.09%) were found to have the highest positivity rate in females (71.43%), along with the confirmed cases of tonsilitis (100%) and hypothyroidism (50%) (Table 2).

### 3.4 Association of covariates with the COVID-19

A binary logistic regression model was performed to examine the adjusted and unadjusted influences (the adjusted odds ratios-AOR and unadjusted odds ratios-UOR) along with the 95% confidence intervals of demographic and health-related factors in the occurrence of corona virus disease (Table 3). Respondent’s residence, gender, age, and symptoms of COVID-19 were found to have a significant association with COVID-19 before or after adjusting the effects of covariates.

**Table 3:** Association of participant’s demographic and clinical characteristics and COVID-19 positivity.

Variables	COVID-19			
	UOR	95% CI	AOR	95% CI
Place of residence				
Gazipur	1.78***	(1.59, 1.98)	1.48***	(1.32, 1.66)
Dhaka	1.00	–	1.00	–
Tangail	3.45***	(2.67, 4.44)	2.48***	(1.90, 3.24)
Others	0.60**	(0.41, 0.88)	0.73	(0.49, 1.08)
Gender				
Female	1.00	–	1.00	–
Male	1.47***	(1.30, 1.65)	1.42***	(1.25, 1.61)
Respondent’s age				
0–20 years	1.00	–	1.00	–
21–40 Years	1.03	(0.85, 1.26)	0.89	(0.72, 1.10)
41–60 Years	1.32**	(1.07, 1.65)	1.22	(0.96, 1.54)
Above 60 Years	2.41***	(1.72, 3.53)	1.95***	(1.36, 2.79)
Symptoms of COVID-19				
No	1.00	–	1.00	–
Yes	3.46***	(3.11, 3.36)	3.22***	(2.88, 3.61)
Comorbidity				
No	1.00	–	1.00	–
Yes	1.69***	(1.42, 2.01)	1.08	(0.88, 1.31)

*p-value < 0.05; **p-value < 0.01; ***p-value < 0.001.

Method: binary logistic regression model was used to observe the adjusted effects of covariates on COVID-19. Adjusted odds ratios-AOR; Unadjusted odds ratios-UOR; 95% Confidence Interval: CI.

Regarding the geographical distribution of COVID-19 cases, respondents from Tangail had 2.48 times (AOR: 2.48, 95% CI: 1.90, 3.24), and from Gazipur had 1.48 times (AOR: 1.48 and 95% CI: 1.32, 1.66) more likelihood to be positive for COVID-19 compared to respondents from Dhaka regions. Older people had a higher risk of COVID-19 as the analysis depicted that individuals with 60 or older age had 95% (AOR: 1.95, 95% CI: 1.36, 2.79) higher odds of being COVID-19 positive compared to the individual with the age of below 20 years old (Table 3).

Male individuals were 42% (AOR: 1.42, 95% CI: 1.25, 1.61) more likely to be affected by COVID-19 compared to females. Furthermore, the respondents with symptoms had 3.22 times more likelihood to suffer from COVID-19 (AOR: 3.22, 95% CI: 2.88, 3.61) than the asymptomatic individuals (Table 3). The effect of comorbidity on COVID-19 was found to be significant when the effects of other factors were not controlled. However, there is no significant association between comorbidity and COVID-19 exposure when the effects of other covariates were considered (Table 3).

## 4 Discussion

Over the last 50 years, several corona virus species have introduced various human and livestock diseases. Due to their recombination capability, random mutation, and multiple species infection ability, new variants continue to strike affecting human health. The novel SARS-CoV-2 corona virus has threatened human life and global health, causing the COVID-19 pandemic since December 2019. The report has focused on investigating the various aspects of this viral infections and the clinical outcome on key epidemiological parameters by interpreting the clinical history of every individual who has given samples for the COVID-19 test at the laboratory during the nationwide lockdown situation in Bangladesh.

The pre-requisite condition to reopen the readymade garments companies for their regular production and exportation process, was to ensure a COVID-19 negative office environment (35, 36). We established the laboratory exclusively for the detection of COVID-19, and diagnosed random samples from a large population (*N* = 8480) from several garments and industries around the EPZ (Dhaka Export Processing Zones) at Gazipur for occupational health and safety purposes. As per the CDC guideline, we enlisted the current residence address of every individual (33). We found many of them became infected from their working environment and spread the virus to their families. Hence, all the samples were marked as human-transmitted and mostly inhabited in the cluster zone of Dhaka region, the capital of Bangladesh. The IEDCR also published surveillance showing that 45% of dwellers of Dhaka had been exposed to COVID-19 by October 2020 (37).

Globally, the chronological age is considered a highly significant predictor in measuring disease severity, future disease occurrence assumption, and reason for death rates (22, 23); although the biological age plays a potential role, especially two biomarkers-the epigenetic and glycan clock (21). Glycan regulates many immunological pathways modified with different age ranges, which are most susceptible to SARS-CoV-2 attachment. The primary receptor of SARS-CoV-2, ACE II is highly glycosylated; even the ABO blood group system is based on the diversity of the glycan molecules (22). This could be a predominant reason for our study to observe the highest COVID-19 positivity rate at the age range of more than 60 years old, compared to the other three age range categories (Table 1), although the minimal number of total cases (2.58%) in the present study.

Similarly, gender influenced viral infection effectively, more than two-fold higher in men than women, where biological factors could impact the total number of cases, hospitalization, duration of recovery, and fatal rate (38). One of the most fundamental biological factors is the activation of the sex hormones, especially estrogen, which influences both the innate and adaptive immune response in females faster than in males despite being highly susceptible to autoimmune diseases (24). We have also observed the same difference in our analysis: two-thirds of samples were collected from males, and the prevalence of COVID-19 was 2.74 times higher than in females. The immunization schedule started precisely 8 months after the establishment of our laboratory, albeit people were frightened to receive vaccines for the post-vaccination complications. Thus, we inscribed only 2.03% of vaccination information from the enrolled population.

The vital principle of our laboratory was early monitoring of random people with or without symptoms as per the requirement of rejoining to the workplaces and being socialized by maintaining accurate health and safety protocol (36). Therefore, we have collected two-thirds of asymptomatic samples and successfully diagnosed a significant number of cases as SARS-CoV-2 positive (13.96%) at the latent period. Conversely, one-third of symptomatic individuals were reported as positive (35.96%), although this was the most remarkable indicator of SARS-CoV-2 susceptibility. The rest of the two third symptomatic people probably expressed post-COVID-19 complications. Fever and cough were the most commonly reported symptoms among all ages.

Our laboratory and the designated hospital were not authorized for critically ill patients, or as the COVID-19 support center. Therefore, we received a limited number of samples with a history of chronic underlying diseases and estimated the prevalence to evaluate it as an essential co-factor. One-third of individuals with comorbiddities had a likelihood of being COVID-19 positive, while other factors were not adjusted statistically. Interestingly, three-fourths of individuals with pre-medical history were male, highlighting diabetes mellitus, hypertension, CVD, renal disease, and asthma. Alternatively, autoimmune disease and cancer were prevalent in the case of female individuals, similarly as explained in other study (39).

Our study enrolled a large, healthy Bangladeshi population with diversified responses in susceptibility to the novel virus SARS-CoV-2 in the first year of the pandemic (2020–21). The objective of this research was to explore COVID-19 through the general public with great concern in the restricted environment of nationwide lockdown. Therefore, the limitation of this study was not being able to follow up with every individual for continuous data collection in case of hospitalization or discharge dates, recovery, and mortality rate, as our hospital was not an isolation center. Several individuals rarely could have remembered any symptoms at the onset before the test. Thus, we could not estimate the duration of the incubation period to the length of long COVID-19 cases. Our laboratory work was circumscribed for pediatric and sensitive patient sample collection rather than the pedestrians that affect the age range being heteroscedastic in regression analysis. Moreover, many people were illiterate and unable to mention their regular drug intake related to specific comorbidities that might interfere with the test result. Overall, the result provides a broad range of epidemiological analysis that would be simultaneously supportive for future experiments of drug and vaccine designing of new variants of SARS-CoV-2 for the people of the South Asian region.

## Data availability statement

The raw data supporting the conclusions of this article will be made available by the authors, without undue reservation.

## Ethics statement

The studies involving humans were approved by ERC of Department of Biochemistry and Molecular Biology, University of Dhaka, Bangladesh. The studies were conducted in accordance with the local legislation and institutional requirements. The participants provided their written informed consent to participate in this study.

## Author contributions

MH: Conceptualization, Project administration, Supervision, Writing – original draft, Writing – review & editing. RM: Data curation, Formal analysis, Investigation, Project administration, Writing – original draft. SI: Writing – original draft, Writing – review & editing. LB: Writing – original draft, Writing – review & editing. AJ: Formal analysis, Software, Writing – original draft, Writing – review & editing. ZH: Project administration, Supervision, Writing – original draft. SE: Supervision, Writing – original draft. SD: Project administration, Supervision, Writing – original draft. AA: Project administration, Supervision, Writing – original draft, Writing – review & editing.
